# Efficacy and Safety of Pulsed Field Ablation in Atrial Fibrillation: A Systematic Review

**DOI:** 10.3390/jcm12020719

**Published:** 2023-01-16

**Authors:** Jurgen Shtembari, Dhan Bahadur Shrestha, Bishnu Deep Pathak, Bishal Dhakal, Binit Upadhaya Regmi, Nimesh K. Patel, Ghanshyam Palamaner Subash Shantha, Gautham Kalahasty, Karoly Kaszala, Jayanthi N. Koneru

**Affiliations:** 1 Department of Internal Medicine, Mount Sinai Hospital, Chicago, IL 60608, USA; 2 Department of Internal Medicine, Jibjibe Primary Health Care Center, Jibjibe 45000, Nepal; 3 Department of Internal Medicine, Nepalese Army Institute of Health Sciences, Kathmandu 44600, Nepal; 4 Department of Internal Medicine, Division of Cardiology, School of Medicine, Virginia Commonwealth University, Richmond, VA 23219, USA; 5 Department of Internal Medicine, Division of Electrophysiology, Atrium Health, Wake Forest Baptist Health, Medical Center Boulevard, Winston-Salem, NC 27157, USA; 6 Department of Internal Medicine, Division of Electrophysiology, School of Medicine, Virginia Commonwealth University, Richmond, VA 23219, USA

**Keywords:** atrial fibrillation, pulmonary vein isolation, catheter ablation, pulse-field ablation

## Abstract

Background: Atrial fibrillation (AF) is the most common cardiac arrhythmia associated with high morbidity and mortality. AF treatment is guided by a patient–provider risk–benefit discussion regarding drug versus ablation or combination. Thermal ablation has a high rate of adverse events compared to pulsed field ablation (PFA). In this systematic review, we aimed to determine the safety and efficacy of PFA. Methods: The electronic search for relevant articles in English was completed in PubMed, PubMed Central, Cochrane library, Scopus, and Embase databases till July 2022. The screening was completed via the use of Covidence software. The risk of bias assessment and data extraction from the included studies was performed, and the narrative synthesis was performed accordingly. Results: A total of six studies were selected for review and 1897 patients receiving PFA were involved in these studies. Our review was focused on pulmonary vein isolation success, major adverse events, and arrhythmia recurrence. Successful pulmonary vein isolation (PVI) was completed in 100% of cases except in two studies. In one of them, six out of seven patients (86%) in the epicardial cohort had successful PVI. In the MANIFEST-PF survey, the acute PVI success rate was 99.9%. The major complications were rare and included pericardial tamponade, vascular complications requiring surgery, and stroke. The atrial arrhythmia recurrence was higher in the thermal group than in the PFA group (39% vs. 11%). Conclusions: The success rate of PVI by PFA is high, and major adverse events are low. PFA is found to decrease the recurrence of atrial arrhythmia compared to thermal ablation. Substantial randomized controlled trials (RCTs) are needed to validate the efficacy and safety of PFA over conventional methods.

## 1. Introduction

Atrial fibrillation (AF) is the most common clinically significant cardiac arrhythmia. Its prevalence is increasing worldwide [1,2]. The lifetime risk of developing AF beyond 40 years of age is 26% and 23% for males and females, respectively. Hypertension, obesity, obstructive sleep apnea, alcohol consumption, and thyroid disorders are some of the modifiable risk factors. Age is the most important non-modifiable risk factor in AF [3]. The research has shown that prevalence roughly doubles with each decade of life [4].

AF is associated with a higher risk of morbidity and mortality from cardiovascular events (heart failure, myocardial infarction, and sudden cardiac death), thromboembolism, ischemic stroke, and renal disease [2,4,5].

To date, pulmonary vein isolation (PVI) is predominantly performed through conventional thermal methods that include radiofrequency, cryotherapy, laser, and ultrasound. However, it has been well-studied that these methods are associated with indiscriminate tissue damage leading to esophageal, phrenic nerve, and aortic injuries [6,7,8]. The thermal methods work by inducing coagulative necrosis and subsequently reparative fibrosis, which may result in pulmonary vein stenosis and impaired left atrial reservoir function [9]. In contrast, pulsed field ablation (PFA) is a new approach to cardiac ablation of AF. It employs a non-thermal ablative mechanism in which cell death is obtained by applying ultra-short electrical pulses to induce pores in cell membranes [6,10]. It has higher myocardial tissue selectivity compared to conventional methods. PFA ablation is effective for paroxysmal and persistent AF [7,11] and is associated with low AF recurrence at one-year follow-up [12].

A few clinical studies have reported higher durability and safety profile of PFA over conventional methods of ablation [6,11,13]. PFA lesions are homogenous with an intact extracellular matrix structure, nerves, and microvasculature [9]. More experimental studies on the efficacy and safety profile of PFA for AF are ongoing, and further evidence is yet to be explored. This study aims to review the efficacy and safety of PFA and compare it with conventional ablation methods.

## 2. Materials and Methods

The study protocol has been registered in the International Prospective Register of Systematic Reviews (PROSPERO ID: CRD42022325651). The Preferred Reporting Items for Systematic Reviews and Meta-Analysis (PRISMA) guidelines were followed while conducting this study.

### 2.1. Criteria for Considering Studies for This Review

#### 2.1.1. Types of Studies

We systematically analyzed the studies on PFA for atrial fibrillation, with or without comparison with other conventional ablation modalities. These included randomized and non-randomized controlled trials, case–control studies, cohort studies, and cross-sectional studies.

#### 2.1.2. Types of Participants

The patients with atrial fibrillation (paroxysmal or persistent) undergoing ablation procedures were included in our review. Those who were treated with PFA were included in the intervention group, and/or those with conventional methods of ablation such as radiofrequency, cryotherapy, and laser therapy were included in the comparator group.

#### 2.1.3. Types of Interventions

Patients with AF were treated with PFA, with or without a control group (receiving conventional ablation).

#### 2.1.4. Types of Outcome Measures

The studies that reported any of the outcomes of interest (successful pulmonary veins isolation, total procedure time, total fluoroscopy time, acute adverse events, arrhythmia recurrence at follow-up mortality) were included in the review.

### 2.2. Outcomes

The primary outcomes of interest in our review were successful pulmonary vein isolation, acute adverse events, and arrhythmia recurrence at follow-up.

### 2.3. Search Methods for Identification of Studies

The literature search was conducted in PubMed, PubMed Central, Cochrane Library, Scopus, and Embase databases. We performed the search by using the MeSH terms which included pulsed field ablation, and atrial fibrillation. We included the relevant articles in the English language or other languages but have online English translations available, published since the inception of databases to July 2022.

#### Electronic Searches

The detailed search strategy adopted in our review has been attached as Supplementary File S1.

### 2.4. Selection of Studies

The Covidence systematic review software was used to screen studies and extract data. All the studies from the databases were first screened based on their titles and abstracts by two independent reviewers using this software. The third reviewer resolved the conflicts arising during the selection of the studies. After that, the full-text screening of the selected studies was completed by the same method, and any conflict arising in between was resolved accordingly. Following the full-text review, the required data were extracted from the selected studies for qualitative summary.

### 2.5. Data Extraction and Management

Three researchers independently extracted the data, and they were verified in the presence of the fourth researcher. The extracted data included study details, inclusion/exclusion criteria, demographic, and baseline characteristics of patients, reported interventions and comparison groups, and the outcomes of interest. A table was filled appropriately with the extracted data. As the outcomes reported by included studies were heterogeneous and only one study was comparative among included studies with rest being single arm study; meta-analysis could not be performed so, we limited our study to a systematic review only.

### 2.6. Assessment of Risk of Bias in Included Studies

The risk of bias in non-randomized clinical trials was performed by using the ROBINS-I tool [14]. The overall risk of bias was critical in one trial and serious in the other two trials (Supplementary File Table S1). The Joanna Briggs Institute (JBI) checklist [15] was used to assess the risk of bias in three retrospective observational studies (Supplementary File Table S2).

## 3. Results

### 3.1. Study Selection

**Figure 1:**
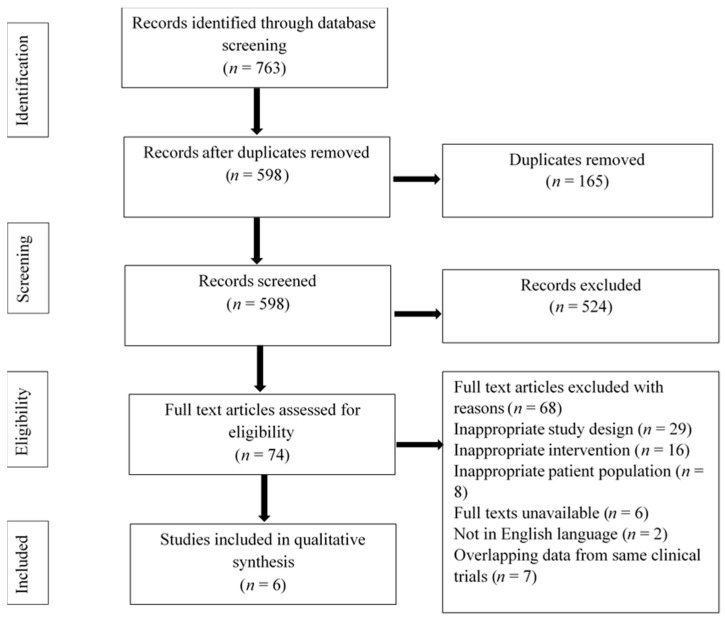
PRISMA flow diagram.

A total of 763 potentially eligible studies were retrieved from the initial database searches. After the removal of 165 duplicates, we screened the title and abstracts of 598 studies. Out of these, 524 articles were excluded due to the unavailability of full texts, and not related to our study objectives. The full texts of 74 studies were assessed, and 68 studies were excluded due to definite reasons (Figure 1). Finally, six studies were included in the qualitative analysis. 

Out of the six studies included, two were single-arm clinical trials, one was a pilot trial, and three were retrospective observational studies. The extracted studies are mentioned in Table 1 and extracted data presented in Table 2. All of them are recent studies conducted after 2018.

### 3.2. Study Population

A total of 1897 patients receiving PFA were involved in six studies. Out of them, MANIFEST–PF survey [18] included the largest number of patients (*n* = 1758 with 34.2% females and 65.8% males). In the rest of the five studies, the total number of participants was 139, of which 92 were males and 47 were females. The age range of the participants was 19 to 92 years across the studies. Most of them had paroxysmal AF, while a few had persistent AF. In a study by Nakatani Y et al., the comparator group receiving thermal ablation comprised 23 patients (males = 17, 74%) with a mean age of 60 ± 8 years.

### 3.3. Successful Pulmonary Vein Isolation

Across five studies, PFA was performed in 1897 patients. Out of them, PVI was successfully completed in 100% of the cases in four studies. In a study by Reddy VY et al. [17], PFA was performed by using an ablation catheter (endocardial cohort, *n* = 15) and by surgical method (epicardial cohort, *n* = 7). In the former cohort, successful PVI was completed in all cases whereas, in the latter group, only six out of seven (86%) patients had successful PVI. Likewise, in the MANIFEST-PF survey [18], the acute PVI success rate was 99.9% (range = 98.9–100).

### 3.4. Timing of Procedure

The total procedure time of PFA varied from 38 min to 215 min across different studies. In the study by Nakatani Y et al. [9], the procedure time for the thermal group was significantly higher than that for the PFA group (130 (110–200) min vs. 96 (77–111) min, *p* = 0.001). In the MANIFEST-PF survey [18], the mean procedure time was 65 min (range 38–215).

Likewise, the time taken for the fluoroscopy procedure in PFA varied from 4.5 min to 33 min across six studies. The maximum time (28 ± 9 min) for this procedure was reported by Verma A et al. [1], whereas the minimum time of 6.6 ± 3.8 min was taken by Reddy VY et al. [17]. The Nakatani’s study [9], the fluoroscopy time for the thermal group was lower than that for the PFA group (20 (18–31) min vs. 23 (17–29) min).

In Reddy’s study [7], total PV isolation time was 22 (15–29) min. Likewise, in the same study, the time for left atrial posterior wall ablation and cavo-tricuspid isthmus ablation was 10 (6–13) min and 9 (6–12) min, respectively. Similarly, in another study by Reddy VY et al. [17], the total ablation time for the endocardial and epicardial cohorts was 19.0 ± 2.5 min and 25.0 ± 17.5 min, respectively.

### 3.5. Adverse Events/Complications

Vascular access site hemorrhage (*n* = 1/38) and cardiac tamponade/perforation (*n* = 1/25) were the serious adverse events reported by Verma A et al. [1] and Reddy VY et al. [7], respectively. In another study by Reddy VY et al. [16], both major and minor complications were observed, which included major vascular complications requiring surgical repair (*n* = 1/76, 1.3%), and minor complications like esophageal erythema (*n* = 2/60, 3.3%) and minor vascular complications (*n* = 4/76, 5.3%). In the study by Nakatani Y et al. [9], the minor complication was groin hematoma in both PFA (*n* = 1, 6%) and thermal groups (*n* = 2, 9%), which resolved on conservative management.

In the MANIFEST-PF survey [18], the major complication rate was 29 (1.6%), which included pericardial tamponade (17, 0.97%), stroke (7, 0.39%), and vascular complications requiring surgery (4, 0.23%). One patient with a stroke died (0.06%). On the other hand, the minor complication rate was 68 (3.86%), which included vascular complications (3.28%), phrenic nerve injury (0.46%), and transient ischemic attack (0.11%). Other rare, unusual events observed were intraprocedural coronary artery spasm, hemoptysis, and dry cough persistent for six weeks, with a rate of 0.06% each.

### 3.6. Recurrence of AF

Likewise, over a comparable follow-up duration (9±3 months vs. 9 ± 4 months, *p* = 0.972), atrial arrhythmia recurred in two (11%) and nine (39%) cases in PFA and thermal groups, respectively. Hence, the arrhythmia-free survival rate was seen to be higher in the PFA group compared to the thermal group (log-rank, *p* = 0.098) [9].

## 4. Discussion

The review was conducted primarily to delineate the therapeutic safety and efficacy of PFA as compared to other conventional ablative methods in patients undergoing an ablative procedure for AF. The efficacy and safety of PFA were determined in terms of successful PVI, the occurrence of adverse events or outcomes, and the recurrence of AF or other arrhythmias. For this, we included six studies of which, two were single-arm clinical trials, one was a pilot trial, and three were retrospective observational studies. MANIFEST-PF survey was a multinational retrospective survey conducted in 24 clinical centers across the world by incorporating the maximum number of patients (*n* = 1758).

The comparator group included ablative methods performed by either radiofrequency, cryotherapy, laser therapy, or ultrasound. However, among six studies included in the review, only one study by Nakatani et al. [9] had the comparator group. From all the pooled studies, the major outcomes that have contributed to the safety and efficacy of PFA in AF are discussed here.

### 4.1. Successful Pulmonary Vein Isolation (PVI)

Pulmonary vein isolation (PVI) is one of the major determining factors for the efficacy of PFA [8,11,13]. Among the six studies, 100% successful PVI was reported in four studies. However, in a study by Reddy VY et al. [17] where PFA was performed by ablation catheter (endocardial cohort, *n* = 15) and surgical method (epicardial cohort, *n* = 7), 100% successful PVI was performed only in the endocardial cohort (15 out of 15). The epicardial cohort, where PFA was carried out by surgical means, had 86% (six out of seven) successful PVI. The remaining unsuccessful PVI was attributed to the system’s failure to deliver PFA appropriately due to technical problems. As for that, the patients (six out seven) who received successful delivery of pulsed electric field (PEF) pulses had pulmonary veins isolated in all of the cases. In addition to PVI, Reddy VY et al. [17] reported successful left atrial posterior wall (LAPW) isolation in all of the cases (six out of seven) who received successful delivery of PEF pulses. Similarly, successful LAPW ablation (24 out of 24 patients) and cavo-tricuspid isthmus block (100% on both studies by Reddy VY et al.) are reported by Reddy VY et al. [7,16]. The durability of LAPW (100%, *n* = 21 of 21) and PVI (96%, 82 of 85 PVs) lesions was reported by Reddy VY et al. [7]. Likewise, the success rate of PVI in MANIFEST-PF was 99.9%. This points towards the efficacy of PFA in producing successful ablative lesions in AF patients.

### 4.2. Adverse Events/Outcomes

The common adverse events due to PFA that have been described include esophageal injury, phrenic nerve palsy, aortic injury, vascular access site hemorrhage, cardiac tamponade/perforation, stroke, myocardial infarction, heart block, pericarditis, death, atrioesophageal fistula, and PV stenosis/narrowing [1,7,9,16,17]. Groin hematomas were the complications observed in the study by Nakatani et al. [9] where the comparator was a thermal group. Of the groin hematomas, one of them happened in the PFA group (*n* = 18) and two in the thermal group (*n* = 23). They were conservatively managed without requiring any surgical intervention. On cardiac magnetic resonance (CMR) imaging, signs of intramural hemorrhage or microvascular damage were seen in none of the patients after PFA as compared to the thermal group (PFA vs. thermal group, *p* < 0.001) where such signs (mix of hyper-enhanced and dark areas) were seen in all patients. Similarly, acute tissue edema was slightly less in PFA than in the thermal group (10.0 ± 1.5 mL vs. 12.0 ± 2.1 mL, *p* = 0.002). In the chronic stage, the acute late gadolinium enhancement (LGE) seen on CMR had disappeared in the majority of the PFA group (mean LGE reversibility 60 (55–65)% of acute values). However, this reversible change was seen much less in the thermal group (18 (12–34)%, *p* < 0.001 vs. PFA). This finding ensures tissue safety by PFA as compared to a conventional method such as thermal ablation. This is evidenced by the preservation of the extracellular matrix framework and the lack of provoking inflammatory reactions by PFA [19,20,21]. The effect on left atrium (LA) structure and function was shown by evidence of LA fibrosis (16.7 ± 3.4% vs. 17.3 ± 3.7%, PFA vs. thermal group) and declined LA ejection fraction (58 (48–66)% vs. 55 (41–65)%, PFA vs. thermal group) in cine images of CMR. The decline in LA ejection fraction was less in the PFA group with a lower percentage of LA fibrosis as compared to the thermal group. Finally, the wall compliance, which declined acutely in both PFA and thermal ablation recovered only with PFA in the chronic stage. This led to the recovery of the left atrium reservoir and booster pump functions which is suggestive of PFA preserving the LA kinetic function.

In the study by Verma et al. [1], no serious adverse events occurred in any patient (*n* = 38) in 30 days post-procedure follow-up. However, one vascular access site hemorrhage was reported as a procedure-related event. Similarly, as described by Verma et al. the duration of energy application in PFA is shorter as compared to sustained energy application in the thermal ablative procedure. This signifies the efficiency of PFA compared to other conventional ablative procedures such as thermal ablation.

The study by Reddy VY et al. [7] reported only one adverse event, cardiac tamponade/perforation, out of a total of 25 patients (1/25, 4%). The explanation behind this event was that it occurred during remapping using radiofrequency only. The post-procedure esophagogastroduodenoscopy and repeat cardiac computed tomography revealed no mucosal lesions or PV narrowing, respectively. Likewise, there were no esophageal lesions and phrenic nerve paresis/palsy. However, due to a single occurrence of inadvertent transient acute left atrial appendage (LAA) isolation with subsequent recovery, it warrants careful assessment of ablation catheter and spline positioning when in proximity to such critical structures.

In another study by Reddy VY et al. [16], a total of five vascular complications (one major left groin hematoma and four minor groin hematomas) were reported (*n* = 76) with a vascular complication rate of 6.6%. Additionally, the primary safety endpoint rate was 1.3% i.e., one out of seventy-six patients requiring surgical revision. There were no instances of pericardial tamponade, phrenic nerve palsy, PV stenosis, stroke, atrioesophageal fistula, or death. Similarly, there were no device-related complications, except minor mucosal thermal injury in two of thirty-six patients where PFA and RFA were performed posteriorly and anteriorly,, respectively. The post-procedure MRI brain in 51 of 76 (67%) patients at 1.2 ± 0.6 days revealed silent cerebral events and cerebral lesions in 5 (9.8%) and 3 (5.9%) patients, respectively. However, all the lesions were asymptomatic. Furthermore, cardiac computed tomography at acute post-ablation and 75 ± 11 days (*n* = 44 patients) revealed no evidence of PV stenosis.

In another study by Reddy VY et al. [17], no adverse events during the procedure or post- procedure as stated above were reported (*n* = 22). Likewise, there was no evidence of malignant arrhythmias, significant electrocardiographic repolarization changes, and ventricular repolarization occurrence. Additionally, at the one-month follow-up, no adverse events were reported.

The major adverse events such as cardiac tamponade and vascular complications requiring surgery were comparatively lower in the MANIFEST-PF survey [18]. This could be explained by the very large study population of this survey which seems to be more representative of a larger population. However, some unusual complications were also reported by this survey that included intraprocedural coronary artery spasm, hemoptysis, and dry cough persistent for six weeks. One of the limitations of this study is that it only explained the acute cases but did not report the recurrence of arrhythmia post-ablation.

### 4.3. Recurrence of AF or Other Atrial Arrhythmias

Out of the five studies, Nakatani et al. [9] described the recurrence of AF or any other atrial arrhythmias over a comparable follow-up duration (9 ± 3 months vs. 9 ± 4 months, PFA vs. thermal group) for the PFA and thermal group. The atrial arrhythmia recurred in 2 (11%) and 9 (39%) cases in PFA and thermal groups over that duration, respectively. This led to the finding that the arrhythmia-free survival rate was higher in the PFA group as compared to the thermal group (log-rank, *p* = 0.098). Although the remaining four studies mentioned no adverse events in the follow-up, the recurrence of AF or any other atrial arrhythmias was not highlighted.

## 5. Limitation 

In this manuscript, we attempted to evaluate the safety and efficacy of PFA to other modalities of AF ablation. We systematically searched major databases and thoroughly reviewed the published literature. We included six studies; three of them are RCTs and the rest are observational cohort studies. All these studies are relatively small except MANIFEST-PF; therefore, MANIFEST-PF carries 92.7% of the weightage of the total included patient population. Given the limited availability of comparative data, and heterogeneous studies in terms of setting, procedural techniques, device, and size of studies, we did not perform a meta-analysis. At last, some of the findings reported in our review were reported by small-sized non-randomized studies so, further, bigger comparative RCTs with longer-term follow-up are required to assure the safety and superiority of the PFA over other ablation techniques in the AF population.

## 6. Conclusions

The success rate of PVI by PFA is high, and major adverse events are low. PFA is found to decrease the recurrence of atrial arrhythmia compared to thermal ablation. Substantial randomized controlled trials (RCTs) are needed to validate the efficacy and safety of PFA over conventional methods.

## Figures and Tables

**Table 1:** Number of participants in included studies.

Studies	Total Patients Treatedwith PFA Only (*n*)	Males	Females
Verma A et al. [1]	38	20	18
Reddy VY et al. [7]	25	20	5
Reddy VY et al. [16]	36 (PF/PF cohort)	25	11
Reddy VY et al. [17]	22	12	10
Nakatani Y et al. [9]	18	15	3
Ekanem E et al. [18]	1758	65.8%	34.2%

**Table 2:** Narrative summary of included studies.

Study ID	Study Type	Study Population	Intervention	Comparator	Outcomes
Verma A et al., [1] 2022	PULSED AF pilot trial	*n* = 38 Males = 20 (53%) Age = 62.0 ± 11.3 years AF type = paroxysmal or persistent	PFA system delivering bipolar, biphasic electric fields through a circular multielectrode array catheter.	None	Successful PVI = 100% Total procedure time = 160 ± 91 min fluoroscopy time = 28 ± 9 min Device left atrial dwell time = 82 ± 35 min Electrode temperature rise = 2.1 ± 2.2 °C vascular access site hemorrhage = 1/38 Phrenic nerve injury/diaphragmatic paralysis at 30 days post-ablation = 0 atrioesophageal fistula = 0 Cardiac adverse events = 0 Mortality = 0
Reddy VY et al., [7] 2020	Clinical trial (PersAFOne clinical trial)	*n* = 25 Age = 67 (60–70) years Male = 20 (80%) AF type = persistent	Biphasic, bipolar PFA using a multispline catheter for both PVI and LAPW ablation	None	Acute PVI = 96/96 (100%) chronic PVI (*n* = 22) = 82/85 (96%) Chronic LAPW isolation (*n* = 22) = 21/22 (95%) Procedure time = 125 (108–166) min Fluoroscopy time = 16 (12–23) min adverse events within 30 days of procedure: Cardiac tamponade/perforation = 1/25 (4%) Late-onset complications (PV stenosis, atrioesophageal fistula) = 0
Reddy VY et al., [16] 2020	Clinical trial (single-arm, multi-center)	*n* = 76 (out of these, 36 received PFA only) Age = 60.7 ± 8.9 years	PFA with a lattice-tip catheter delivering biphasic PF waveform over 3 to 5 s	None	Successful PVI = 36/36 (100%) fluoroscopy time = 2.7 ± 2.4 min energy application time for: ▪ PVI = 3.2 ± 0.9
		Males = 25/36 (69%)	With total current delivery between 24 and 32 amperes		▪ Mitral isthmus line = 0.9 ± 0.6 ▪ LA roof line = 0.4 ± 0.2 adverse events (*n* = 24): ▪ Esophageal abnormality = 0/24 (0%)
Reddy VY et al., [17] 2018	A prospective, open-label randomized trial	*n* = 22 Endocardial cohort: *n* = 15 Age = 63.8 ± 4.6 yrs Males = 7/15 (46.6%) Epicardial cohort: *n* = 7 Age = 69.0 ± 6.4 yrs Males = 5/7	PFA using a custom over-the-wire endocardial catheter for percutaneous trans-septal PVI, and a linear catheter for encircling the PVs and posterior left atrium during concomitant cardiac surgery	None	For endocardial cohort: ▪ Isolation success = 15/15 (100%) ▪ Procedure time = 67.0 ± 10.5 min ▪ Ablation time = 19.0 ± 2.5 min ▪ Fluoroscopy time = 12.3 ± 4.0 min For epicardial cohort: ▪ Isolation success = 6/7 (86%) ▪ Ablation time = 25.0 ± 17.5 min ▪ Fluoroscopy time = 6.6 ± 3.8 min
Nakatani Y et al., [9] 2021	Retrospective observational (from IMPULSE and PEFCATtrials)	*n* = 41 Intervention group: ▪ *n*= 18 ▪ Age = 56 ± 9 years ▪ Males = 15 (83%) Comparator group: ▪ *n* = 23 ▪ Age = 60 ± 8 years Males = 17 (74%)	PFA with 12-Fr over- the-wire PFA application catheter with five splines in a flower petal or basket configuration providing monophasic or biphasic pulses	RF ablation (*n* = 16) Cryoablation (*n* = 7)	Intervention group: Successful PVI = 18/18 (100%) total procedure time = 96 (77–111) min Total ablation time = <1 min fluoroscopy time = 23 (17–29) min Complication = 1 (6%) PV reconnection at 3 months remap = 0 Arrhythmia recurrence at (9 ± 3) months= 2 (11%) Comparator group: Successful PVI = 23/23 (100%) Total procedure time = 130 (110–200) min Total ablation time = RF 37(26–72) min, CRYO 16(15–20) min Fluoroscopy time = 20 (18–31) min Complication = 2 (9%) PV reconnection at 3 months remap = NA Arrhythmia recurrence at (9 ± 4) months= 9 (39%)
Ekanem E et al., [18] 2022	A retrospective observational study (multinational survey from 24 clinical centers; named as MANIFEST- PF survey)	*n* = 1758 Males = 65.8% Age = 61.6 (19–92) years	PFA via 12-Fr over-the-wire pentaspline catheter (Farawave) applied in basket and flower configuration for PFA, and in flower configuration for posterior left atrial wall ablation	None	PVI success rate = 99.9% (range = 98.9–100) Total procedure time = 65 (38–215) min Fluoroscopy time = 13.7 (4.5–33) min Major adverse events: ▪ Pericardial tamponade (17, 0.97%) ▪ Stroke (7, 0.39%) ▪ Vascular complications (4, 0.23%) ▪ Coronary artery spasm (1, 0.06%) Minor adverse events: ▪ Vascular complications (3.28%) ▪ Phrenic nerve injury (0.46%) ▪ Transient ischemic attack (0.11%) mortality = 1 (0.06%)

PV: pulmonary vein; PVI: pulmonary vein isolation, LA: left atrium; LAPW: left atrium posterior wall; min: minutes; PFA: pulsed filed ablation; *n*: number; yrs: years.

## Data Availability

The data analyzed during the current study are available within the manuscript or in supplementary files.

## Supplementary Materials

The following supporting information can be downloaded at: https://www.mdpi.com/article/10.3390/jcm12020719/s1. Supplementary file S1. Electronic Search Details; Table S1. Risk of bias assessment by using ROBINS-I tool. Table S2. Risk of bias assessment by using JBI checklist.
