# Supplementary material for: Efficacy and Safety of Pulsed Field Ablation in Atrial Fibrillation: A Systematic Review
Source: J Clin Med. 2023 Jan 16;12(2):719. doi: 10.3390/jcm12020719 (PMC9861350; doi:10.3390/jcm12020719)
Supplement: Supplementary file 1 [file jcm-12-00719-s001.zip › jcm-2154330-supplementary.pdf]

Supplementary Materials

***Supplementary File** S1. Electronic Search Details*

Search builder: (Pulsed field ablation) AND (atrial fibrillation)

PubMed. Link: https://pubmed.ncbi.nlm.nih.gov/?te\linebreakrm=%28Pulsed+field+ablation%29+AND+%28atrial+fibrillation%29&sort=relevance

Hits: 135

PubMed Central. Link: https://www.ncbi.nlm.nih.gov/pmc/?term=(Pulsed+field+ablation)+AND+(atrial+fibrillation)

Hits: 418

Scopus. Link: https://www.scopus.com/results/results.uri?sort=plf-f&src=s&st1=%28Pulsed+field+ablation%29+AND+%28atrial+fibrillation%29&sid=f21f41e0a6a75e7aaf95573a0207ece3&sot=b&sdt=b&sl=64&s=TITLE-ABS-KEY%28%28Pulsed+field+ablation%29+AND+%28atrial+fibrillation%29%29&origin=searchbasic&editSaveSearch=&yearFrom=Before+1960&yearTo=Present

Hits: 83

Cochrane Library. Link: https://www.cochranelibrary.com/advanced-search

Hits: 8.

Clinical trial registry. Link: https://www.clinicaltrials.gov/ct2/results?cond=Atrial+Fibrillation&term=Pulsed+field+ablation&type=&rslt=&age_v=&gndr=&intr=&titles=&outc=&spons=&lead=&id=&cntry=&state=&city=&dist=&locn=&rsub=&strd_s=&strd_e=&prcd_s=&prcd_e=&sfpd_s=&sfpd_e=&rfpd_s=&rfpd_e=&lupd_s=&lupd_e=&sort=

Hits: 12

Embase. Link: https://www.embase.com/#advancedSearch/resultspage/history.3/page.1/25.items/orderby.date/source.

Hits: 119

Table S1. Risk of bias assessment by using ROBINS-I tool.

SN	Domains	Reddy VY et al [1]	Reddy VY et al [2]	Verma A et al [3]
1	Bias due to confounding	Serious	Serious	Serious
2	Bias in selection of participants into the study	Low	Low	Low
3	Bias in classification of interventions	Low	Low	Low
4	Bias due to deviation from intended interventions	Low	Low	Low
5	Bias due to missing data	Low	Low	Low
6	Bias in measurement of outcome	Moderate	Critical	Serious
7	Bias in selection of the reported result	Serious	Moderate	Low
	Overall risk of bias	Serious	Critical	Serious

Table S2. Risk of bias assessment by using JBI checklist.

		Nakatani Y et al [4]	Reddy VY et al [5]	Ekanem E et al [6]
1.	Was the sample frame appropriate to address the target population?	No	No	No
2.	Were study participants sampled in an appropriate way?	No	No	No
3.	Was the sample size adequate?	Not applicable	Not applicable	Unclear
4.	Were the study subjects and the setting described in detail?	Yes	Yes	Yes
5.	Was the data analysis conducted with sufficient coverage of the identified sample?	Yes	Yes	Yes
6.	Were valid methods used for the identification of the condition?	Yes	Yes	Yes
7.	Was the condition measured in a standard, reliable way for all participants?	Yes	Yes	Yes
8.	Was there appropriate statistical analysis?	Yes	Yes	Yes
9.	Was the response rate adequate, and if not, was the low response rate managed appropriately?	Yes	Yes	Yes

References:

[1] Reddy, V.Y.; Anter, E.; Rackauskas, G.; Peichl, P.; Koruth, J.S.; Petru, J.; Funasako, M.; Minami, K.; Natale, A.; Jais, P.; et al. Lattice-Tip Focal Ablation Catheter That Toggles Between Radiofrequency and Pulsed Field Energy to Treat Atrial Fibrillation: A First-in-Human Trial. *Circ. Arrhythmia Electrophysiol.* **2020**, *13*, 483–495. https://doi.org/10.1161/CIRCEP.120.008718.

[2] Reddy, V.Y.; Koruth, J.; Jais, P.; Petru, J.; Timko, F.; Skalsky, I.; Hebeler, R.; Labrousse, L.; Barandon, L.; Kralovec, S.; et al. Ablation of Atrial Fibrillation With Pulsed Electric Fields: An Ultra-Rapid, Tissue-Selective Modality for Cardiac Ablation. *JACC Clin. Electrophysiol.* **2018**, *4*, 987–995. https://doi.org/10.1016/J.JACEP.2018.04.005.

[**3**] Verma, A.; Boersma, L.; Haines, D.E.; Natale, A.; Marchlinski, F.E.; Sanders, P.; Calkins, H.; Packer, D.L.; Hummel, J.; Onal, B.; et al. First-in-Human Experience and Acute Procedural Outcomes Using a Novel Pulsed Field Ablation System: The PULSED AF Pilot Trial. *Circ. Arrhythmia Electrophysiol.* **2022**, *15*, e010168. https://doi.org/10.1161/CIRCEP.121.010168.

[4] Nakatani, Y.; Sridi-Cheniti, S.; Cheniti, G.; Ramirez, F.D.; Goujeau, C.; André, C.; Nakashima, T.; Eggert, C.; Schneider, C.; Viswanathan, R.; et al. Pulsed field ablation prevents chronic atrial fibrotic changes and restrictive mechanics after catheter ablation for atrial fibrillation. *EP Eur.* **2021**, *23*, 1767–1776. https://doi.org/10.1093/EUROPACE/EUAB155.

[5] Reddy, V.Y.; Anic, A.; Koruth, J.; Petru, J.; Funasako, M.; Minami, K.; Breskovic, T.; Sikiric, I.; Dukkipati, S.R.; Kawamura, I.; et al. Pulsed Field Ablation in Patients With Persistent Atrial Fibrillation. *J. Am. Coll. Cardiol.* **2020**, *76*, 1068–1080. https://doi.org/10.1016/J.JACC.2020.07.007.

[6] Ekanem, E.; Reddy, V.Y.; Schmidt, B.; Reichlin, T.; Neven, K.; Metzner, A.; Hansen, J.; Blaauw, Y.; Maury, P.; Arentz, T.; et al. Multi-national survey on the methods, efficacy, and safety on the post-approval clinical use of pulsed field ablation (MANIFEST-PF). *EP Eur.* **2022**, *24*, 1256–1266. https://doi.org/10.1093/EUROPACE/EUAC050.
